# Incidence of Impaired Kidney Function Among Adolescent Patients Hospitalized With Anorexia Nervosa

**DOI:** 10.1001/jamanetworkopen.2021.34908

**Published:** 2021-11-22

**Authors:** Evgenia Gurevich, Shelly Steiling, Daniel Landau

**Affiliations:** 1 Department of Nephrology, Schneider Children's Medical Center of Israel, Petach Tikva, Israel; 2 Department of Dietary Services, Schneider Children's Medical Center of Israel, Petach Tikva, Israel; 3 Sackler School of Medicine, Tel Aviv University

## Abstract

**Importance:**

Anorexia nervosa (AN) is a common psychiatric disorder associated with electrolyte imbalances and impaired kidney function, but their incidence and association with disease severity are unknown.

**Objective:**

To analyze kidney function in patients with AN and its association with body mass index (BMI) and physiologic parameters of disease severity.

**Design, Setting, and Participants:**

Single-center retrospective case-control study of recently hospitalized patients with a diagnosis of AN according to *International Classification of Diseases, Ninth Revision*. All patients were aged 9 to 18 years and hospitalized in the general pediatric ward between 2010 and 2019. BMI and estimated glomerular filtration rate (eGFR) were compared with age- and sex-matched controls hospitalized with other diagnoses.

**Main Outcomes and Measures:**

Impaired kidney function was defined as eGFR less than 90 mL/min/1.73 m^2^. Association between eGFR, BMI, minimal heart rate, and free triiodothyronine (T3) levels were determined using logistic regression.

**Results:**

A total of 395 patients were included in the study group (81.6% were female; mean [SD] age, 14.6 [2.2] years; median BMI percentile, 12.3 [IQR, 0.9-42.0]). Impaired kidney function was found in 36.8% (146 of 395). Mean (SD) eGFR decreased during hospitalization in the group with kidney function impairment (admission: 83 [10.9] mL/min/1.73 m^2^; nadir: 79.1 [8.5] mL/min/1.73 m^2^; latest: 97.7 [15.7] mL/min/1.73 m^2^; *P* < .001). Mean (SD) serum creatinine (SCr) to BMI ratio was higher in both anorexia groups compared with controls in impaired kidney function (4.9% [1.0%]), non–impaired kidney function (3.55% [0.84%]); and control groups (2.8 [1.1%]) (*P* < .001). There was no difference in admission BMI between anorexia groups with and without kidney function impairment. Mean (SD) free T_3_ levels (3.5 [0.2] pmol/L vs 4.08 [1.2] pmol/L; *P* < .001) and mean (SD) minimal heart rate (44 [11] beats per min vs 56 [16] beats per min; *P* < .001) were lower and hospital stay was longer (median, 13 [IQR, 6-21] days vs 8 [IQR, 4-19] days; *P* = .03) in the impaired kidney function group. The highest correlation was found between minimal heart rate and minimal eGFR (*R* = 0.53; *P* < .001).

**Conclusions and Relevance:**

Impaired kidney function in patients with AN is common and transiently worsens during hospitalization. SCr values probably underestimate the degree of kidney function impairment in AN. Results of this study found that patients with impaired kidney function had worse anorexia severity parameters unrelated to admission BMI. Therefore, kidney function impairment may be a better marker of anorexia severity.

## Key Points

**Question:**

What is the incidence of impaired kidney function in adolescents with anorexia nervosa, and how does it correlate with body mass index and physiologic parameters of anorexia nervosa severity?

**Findings:**

In this case-control study of 395 adolescent patients with recent diagnosis of anorexia nervosa, impaired kidney function was found in 36.8%. Minimal heart rate and free triiodothyronine levels showed correlation with eGFR but not with admission body mass index.

**Meaning:**

Results of this case-control study suggest that impaired kidney function may be a better parameter of anorexia nervosa severity than body mass index.

## Teaser

This case-control study assesses the prevalence of impaired kidney function in a cohort of pediatric patients with recent diagnosis of anorexia nervosa and examines the association of impaired kidney function with body mass index and disease severity.

## Introduction

Anorexia nervosa (AN) is a common psychiatric disorder that disproportionately affects adolescents and young adults and is associated with high rates of morbidity and mortality. Its complications are associated with the combination of food restriction, weight loss, psychological stress, and the endocrinological and metabolic adaptations to this condition. The reduction of energy intake in patients with AN induces metabolic changes leading to different complications,^1^ including: bradycardia, impaired thyroid function, amenorrhea, and in some cases specific nutritional deficiencies. Kidney-related complications such as electrolyte disturbances, nephrocalcinosis and alterations in water metabolism are common. Acute and chronic kidney disease (CKD) are also described in patients with AN.^2^ Chronic hypokalemia, volume depletion, hypophosphatemia with rhabdomyolysis, and nephrolithiasis are potentially important factors in the development of CKD in patients with AN.^3,4,5^ However, the exact pathophysiologic mechanism of kidney function impairment is not well characterized and the main body of evidence for these phenomena is from studies performed more than 30 years ago.

Previous case series^6,7^ of patients with long-standing AN and severe and irreversible kidney disease have been reported. Kidney histologic findings revealed hypertrophy of the juxtaglomerular apparatus, advanced glomerular collapse, and interstitial fibrosis, consistent with ischemic kidney injury.^6^ In another AN cohort,^7^ end-stage kidney disease was reported in 5.2% of the patients. However, these studies provide data on adults with long-standing (>20 years) AN. The incidence of impaired kidney function (IKF) at the time of AN diagnosis has not yet been determined. We hypothesized that IKF may be an important indicator of AN physiologic severity. The aim of the study was to determine IKF prevalence and its association with disease severity in a cohort of pediatric patients with recent diagnosis of AN.

## Methods

The Rabin-Schneider Institutional Review Board approved the study protocol. We performed a single-center retrospective study of data from all patients aged 9 to 18 years hospitalized in general pediatric wards at Schneider Children's Medical Center in Israel with a recent diagnosis of AN between 2010 and 2019. The ethics committee granted a waiver of informed consent. Hospital medical records were screened for patients with first-time hospitalization with a diagnosis of a*norexia nervosa*, e*ating disorder*, e*ating disorder, unspecified*, l*oss of weight* or b*radycardia* (n = 538). For the latter 2 diagnoses, medical records were investigated, and data from patients with other diagnoses, such as inflammatory bowel disease, cancer, or cardiac conductance abnormalities, were excluded.

Anorexia nervosa was diagnosed according to the criteria of the *Diagnostic and Statistical Manual of Mental Disorders* (Fourth Edition)^8^ (before 2013) and *Diagnostic and Statistical Manual of Mental Disorders* (Fifth Edition) (after 2013).^9^ The diagnosis was based on clinical interviews, patient observation, parental information, and medical evaluations by child psychiatrist or psychologist. Patients with AN are usually referred to a general pediatric ward in the Schneider Children's Medical Center when first diagnosed, for the purpose of acute medical stabilization for a serious decrease in weight or finding of severe bradycardia despite maximally intensive community-based care. Cases with AN hospitalized for other medical conditions were not included in the study group.

The computerized medical record review included the estimated glomerular filtration rate (eGFR), calculated by serum creatinine (SCr) and height using the modified Schwartz equation,^10^ and its correlation to body mass index (BMI) and physiologic parameters of AN severity (free triiodothyronine [T3], nadir nocturnal heart rate, and length of hospitalization). Assuming that eGFR calculation may underestimate the degree of GFR decrease (because of a discrepancy between the preserved height and decreased muscle mass in patients with AN) we also calculated the SCr/BMI ratio. For comparison of normal SCr and SCr/BMI ratio, a randomly selected group of age- and sex-matched patients with normal age- and sex-adjusted SCr values, hospitalized during the same period with other diagnoses (excluding patients hospitalized in oncology and intensive care units) served as controls. The SCr was determined using the enzymatic assay. Missing data on height (n = 103) were approximated, based on the 50th percentile for age and sex, as height should not be depressed in short-term calorically restricted patients in this age range.

### Statistical Analysis

Data were analyzed using BMDP software.^11^ Pearson χ^2^ test or Fisher exact test (2-tailed) were used for analysis of between-group differences in discrete variables, and the analysis of variance was used for continuous variables. Pearson correlations were applied to assess for correlations between variables. Using those variables found to be significant (*P* < .10) on univariate analysis, we applied a stepwise logistic regression to determine those variables significantly associated with eGFR. We used analysis of variance with repeated measures to describe changes in SCr during hospitalization. A *P* value ≤.05 was considered statistically significant. The study followed the Strengthening the Reporting of Observational Studies in Epidemiology (STROBE) reporting guideline for case-control studies.

## Results

A total of 395 individuals were included in the study group and 495 were included in the control group. Mean (SD) age at hospitalization was 14.6 (2.2) years in the study group and 13.6 (2.6) years in the control group; 80.2% of the patients (397 of 495) were girls in the control group and 81.6% (298 of 395) were girls in the group with AN (Table 1). Median BMI percentile on admission was 12.3 (IQR, 0.9-42.0) in the study group vs 49.0 (IQR, 17.0-85.0) in the control group (*P* < .001).

**Table 1.** Comparison Between the Whole Anorexia Nervosa Cohort and Age and Sex Matched Normal Controls^a^

Variable	Mean (SD)		*P* value
	AN whole (n = 395)	Control (n = 495)	
Age, y	14.6 (2.2)	13.5 (2.6)	
Sex, No. (%)			
Female	298 (81.6)	397 (80.2)	
Male	97 (18.4)	98 (19.8)	
BMI percentile, median (IQR)	12.3 (0.9-42.0)	49 (17-85)	<.001
Maximal SCr, mg/dL	0.68 (0.15)	0.54 (0.14)	<.001
SCr/BMI (%)	4 (1.2)	2.8 (1.1)	<.001
Minimal eGFR, mL/min/1.73 m^2^	99.8 (22.0)	124 (26.5)	<.001
IKF, No. (%)	146 (37.0)	0	

Abbreviations: AN, anorexia nervosa; BMI, body mass index; eGFR, estimated glomerular filtration rate; IKF, impaired kidney function (eGFR<90 mL/min/1.73 m^2^); SCr, serum creatinine.

SI conversion factor: To convert creatinine to mmol/L, multiply by 88.42.

^a^ Cases included recently diagnosed AN in patients in their first hospitalization in general pediatric wards for the purpose of acute medical stabilization. Controls were a randomly selected group of age- and sex-matched patients with normal age and sex adjusted SCr values, hospitalized with other diagnoses.

**Figure 1.**
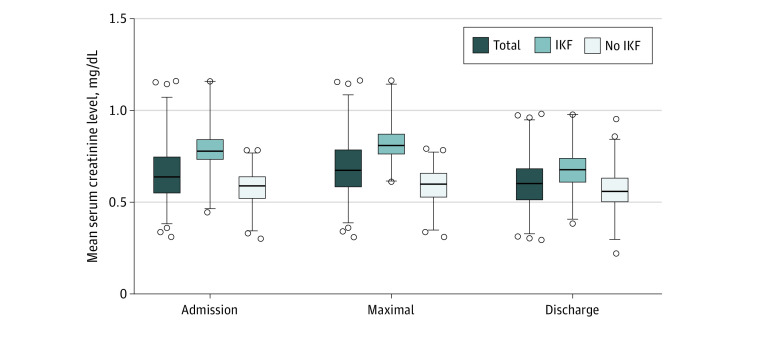
Mean Serum Creatinine Values on Admission, at Maximal Value, and Before Discharge IKF indicates impaired kidney function. SI conversion factor: To convert creatinine to mmol/L, multiply by 88.42.

Median length of hospitalization in AN group was 10 (IQR, 5-20) days, mean (SD) blood pressure (BP) on admission was 102.6 (11.0)/62.7 (9.6) mm Hg and mean (SD) heart rate (HR) on admission was 89 (11) beats per min. Mean (SD) minimal HR was 51.6 (15.8) beats per min. FT_3_ levels were slightly decreased (mean [SD], 3.83 [1.14] pmol/L; normal: 3.1-6.8 pmol/L), with 28% of patients with an FT3 value below normal. Mean (SD) SCr was in the normal range in the entire study group, but was higher in comparison with controls (0.68 [0.15] mg/dL vs 0.54 [0.14] mg/dL: *P* < .001), similar to minimal eGFR (99.8 [22] mL/min/1.73 m^2^ vs 124 [26.5] mL/min/1.73 m^2^; *P* < .001). The mean (SD) SCr significantly increased during hospitalization and then decreased to a lower than admission value in the AN group (admission: 0.66 [0.15] mg/dL, maximal: 0.68 [0.15] mg/dL; discharge: 0.6 [0.13] mg/dL; *P* < .001 by repeated measures analysis of variance) (Figure 1). The SCr/BMI ratio was higher in the AN group compared with controls (4.0% [1.2%] vs 2.8% [1.0%], *P* < .001). Stepwise logistic regression comparing AN cases with controls revealed that SCr/BMI (OR, 3.63; 95% CI, 2.85-4.62; *P* < .001), age (OR, 1.11; 95% CI, 1.04-1.20; *P* < .001), sex (OR, 1.63; 95% CI, 1.05-2.52; *P* = .04), and minimum eGFR (OR, 0.99; 95% CI, 0.98-1.00; *P* = .06) had higher ORs than SCr or eGFR or admission BMI. These parameters yielded a receiver operating characteristics area under the curve of 0.84. Electrolyte abnormalities during hospitalization included hypophosphatemia (<2.5 mg/dL): 5 of 376 (1.3%), hypomagnesemia (<1.7 mg/dL): 1 of 281 (0.35%), hyponatremia (<135 meq/L): 11 of 378 (2.9%), hypokalemia (<3.5 meq/L): 13 of 389 (3.3%) available tests.

For further analysis of kidney function and its association with AN severity, we divided this study cohort into 2 groups according to the minimal eGFR above or below 90 mL/min/1.73 m^2^. The latter (IKF) was found in 36.8% (146 of 395) of patients. The SCr changed substantially during hospitalization (initially increased, then decreased) in both IKF and non IKF groups. However, the mean values were higher in the IKF group (mean [SD] admission 0.79 [0.13] mg/dL, peak 0.83 [0.12] mg/dL, and latest values 0.68 [0.12] mg/dL; *P* < .001) (Figure 1).

Comparison between IKF and non-IKF groups is presented in Table 2. There was no difference in admission median BMI percentile between the groups (13.1 [IQR, 0.5-45] vs 11.7 [IQR, 2.3-35.1]; *P* = .41). A significant difference between IKF and non-IKF groups was found in maximal SCr during hospitalization (mean [SD] 0.83 [0.12] mg/dL vs 0.59 [0.09] mg/dL; *P* < .001). Accordingly, there was a significant difference in mean (SD) minimal eGFR (79.1 [8.5] mL/min/1.73 m^2^ vs 112 [18.5] mL/min/1.73 m^2^; *P* = .001) and SCr/BMI (4.9% [1.0%] vs 3.55% [0.84%]; *P* < .001) between IKF and non IKF groups. Mean (SD) eGFR decreased during hospitalization in the group with IKF for admission (83 [10.9] mL/min/1.73 m^2^) as well as latest (97.7 [15.7] mL/min/1.73 m^2^; *P* < .001). Mean (SD) SCr, eGFR, and SCr/BMI values were not only significantly different between the whole AN group vs controls (Table 1), but also between the non-IKF group vs controls (SCr: 0.59 [0.09] mg/dL vs 0.54 [0.14] mg/dL; *P* < .001; SCr/BMI: 3.6% [0.8%] vs 2.8% [1.1%]; *P* < .001; minimal eGFR: 112 [18.5] mL/min/1.73 m^2^ vs 124 [26.5] mL/min/1.73 m^2^; *P* < .001).

**Table 2.** Comparison Between Impaired Kidney Function and Non–Impaired Kidney Function Subgroups in the Anorexia Nervosa Group

Variable	AN IKF, mean (SD)		*P* value
	Yes (n = 146 [37%])	No (n = 249 [63%])	
Age, y	14.6 (2.1)	14.6 (2.2)	
Sex, No. (%)			<.001
Female	106 (72.6)	215 (86.3)	
Male	40 (27.4)	34 (13.7)	
BMI percentile, median (IQR)	11.7 (2.3-35.1)	13.1 (0.5-45)	.41
Maximal SCr, mg/dL	0.83 (0.12)	0.59 (0.09)	<.001
SCr/BMI, %	4.9 (1)	3.6 (0.8)	<.001
Minimal eGFR, mL/min/1.73 m^2^	79 (8.5)	112 (18.5)	.001
LOH, median (IQR), d	13 (6-21)	8 (4-19)	.03
Minimal HR, bpm	44 (11)	56 (16)	.001
FT_3_, pmol/L	3.5 (0.2)	4.08 (1.2)	.001

Abbreviations: AN, anorexia nervosa; BMI, body mass index; bpm, beats per minute; eGFR, estimated glomerular filtration rate; FT_3_, free triiodothyronine (normal levels, 3.1-6.8 pmol/L); HR, heart rate; IKF, impaired kidney function; LOH, length of hospitalization; SCr, serum creatinine.

SI conversion factor: To convert creatinine to mmol/L, multiply by 88.42.

The FT_3_ levels, available for fewer patients in the AN group (n = 187), were lower in the IKF Vs non IKF group (mean [SD] 3.5 [0.2] pmol/L vs 4.08 [1.2] pmol/L; *P* = .001). A similar difference was seen for minimal mean (SD) HR (44 [11] beats per min vs 56 [16] beats per min; *P* < .001). Length of hospitalization was longer in the IKF group (median 13 [IQR, 6-21] days vs 8 [IQR, 4-19] days; *P* = .03).

We used stepwise logistic regression analysis for the cases group (n = 383), using eGFR above or below 89.9 mL per minute as a cut-off value. We found that SCr/BMI (odds ratio [OR], 8.16 [95% CI, 5.24-12.17]; *P* < .001), minimal HR (OR, 0.96 [95% CI, 0.93-0.98]; *P* < .001), and diastolic and systolic blood pressure on admission independently estimated low vs normal eGFR, yielding an AUC of 0.89.

**Figure 2.**
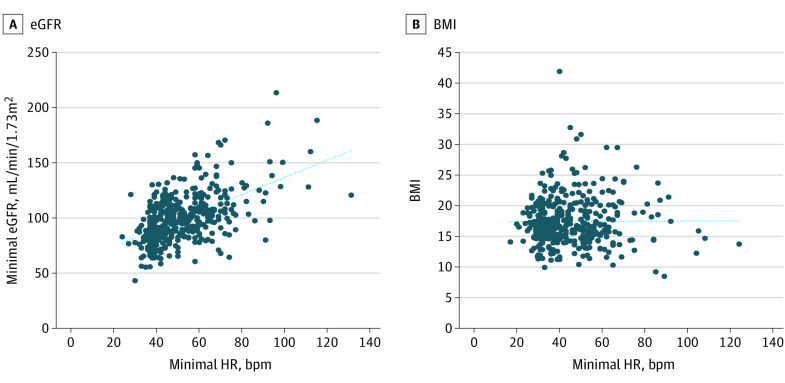
Correlation Between Minimal Heart Rate (HR) vs eGFR or Admission BMI BMI indicates body mass index (calculated as weight in kilograms divided by height in meters squared); eGFR, estimated glomerular filtration rate.

Using Pearson correlation, we found the strongest correlation between minimal HR and minimal eGFR (n = 392; *R* = 0.527; *P* < .001), higher than correlations between minimal HR and SCr/BMI (n = 392; *R* = −0.399; *P* < .001). The FT3 levels also showed a significant correlation with minimal eGFR (n = 83; *R* = 0.34; *P* < .001) similar to SCr/BMI (n = 393; *R* = −0.41; *P* < .001). No correlation between minimal HR and BMI (*R* = 0.02; *P* = .06) or between HR and BMI percentile on admission (*R* = 0.08; *P* = .10) was seen (Figure 2). There was a mild inverse correlation between length of hospitalization to admission BMI percentile (n = 395; *R* = −0.17; *P* < .001).

## Discussion

We describe a large cohort of pediatric patients hospitalized with recent AN diagnosis in one medical center over a period of 10 years. In this cohort, IKF was found in 37% of patients. SCr remained unchanged during hospitalization in the non-IKF group, but increased in the IKF group. Since proper hydration could be assured during hospitalization, this finding suggests the presence of a unique yet undefined mechanism other than dehydration.

To our knowledge, this is the largest cohort of patients with AN with IKF. Previous data exist in cohorts of 14 females^6^ and 45 patients with different types of AN.^12^ Another series reported on long term follow up of 84 patients with AN: 5.2% of them developed end-stage kidney disease and underwent kidney replacement therapy.^7^ As we chose only patients with AN who needed hospitalization, a possible selection bias could exist. Therefore, caution should be used when extrapolating our results to the general AN population.

The GFR calculation usually relies on SCr measurements. As SCr production depends on muscle mass, its level is lower in individuals with reduced muscle mass. Dietary animal protein consumption may also affect serum creatinine concentration.^13^ Patients with AN often have both reduced muscle mass and low dietary animal protein consumption. Therefore, their SCr values are supposed to be lower, compared to individuals with normal muscle mass on a normal diet. In patients with AN, height is supposed to be less affected than weight. Therefore, apparently normal SCr values can lead to overestimation of their renal function, which is based in children on height as the representative surrogate marker of muscle mass. In this AN cohort, mean SCr was in the normal range but higher than in controls. To overcome the potential overestimation of GFR, we describe here data on SCr/BMI ratio as an additional parameter to assess kidney function in such patients. A significant difference in SCr/BMI was found between the whole AN group and controls (Table 1). As may be expected, this difference was more prominent in the IKF group vs controls. However, even in the non-IKF group, with a normal estimated GFR, SCr/BMI was significantly higher compared to controls, hinting for higher SCr values relative to muscle mass in the whole AN group. Thus, normal SCr values in patients with AN may not reflect appropriately kidney function. As serum cystatin C levels reflect eGFR without any dependency on muscle mass,^14^ it could serve as an attractive alternative for this group of patients. This test is not routinely available in Israel. Another alternative way to assess GFR based only on SCr would be the use of the Cockroft Gault formula, which was originally described for adults, but later suggested for adolescents. However, Filler et al^15^ found that the Cockroft Gault formula showed the worst agreement with measured GFR in children aged 1 to 18 years, and therefore is not recommended for use in children.

The exact mechanism of kidney function impairment in AN is not well understood. Nutrition plays a substantial role in renal function.^16^ While GFR increases with animal protein intake, it decreases in a state of protein-energy malnutrition, as well as sodium and acid excretion and urinary concentrating ability.^17^ Healthy patients fed a calorie-deficient diet demonstrate a reduction in creatinine clearance.^18^ Also, healthy patients with decreased protein intake demonstrated a fall in GFR and effective renal plasma flow (RPF), that reversed to normal by protein repletion.^19^ Children with calorie-protein malnutrition demonstrated significant reductions in GFR, RPF and filtration fraction.^20^ Reports on chronic tubulo-interstitial nephropathy in AN attributed it to chronic hypokalemia, acute kidney injury induced by rhabdomyolysis and glomerulosclerosis.^21,22,23^ Histopathologic changes in patients with long-standing AN associated with decreased eGFR and hypokalemia have been reported.^24^ In this cohort, there was no difference in admission BMI between IKF and non-IKF groups, but as mentioned, we describe these patients at the time of AN initial diagnosis in contrast to longer follow up periods described in the literature.

Cardiovascular and endocrinological complications are common in AN, including impaired thyroid function. Total and free triiodothyronine levels are low, as an adaptive mechanism for conserving energy for vital functions, and normalize during weight gain.^25^ Bradycardia and hypotension are cardiovascular responses to starvation due to the profound parasympathetic predominance of low body weights or significant weight loss.^26^ In a retrospective study^27^ of children and teenagers with AN, low GFR was linked to low BMI and bradycardia. Dehydration was not solely responsible for renal impairment.^27^ In this cohort, bradycardia and low FT3 levels were more prominent in the IKF compared to non-IKF group. The strongest correlation was seen between minimal HR and minimal eGFR, higher than correlations between minimal HR vs SCr/BMI. FT3 levels showed a similar correlation with minimal eGFR and SCr/BMI. The length of hospitalization was longer in IKF group than in non-IKF group also indicating IKF as reflecting a more severe AN condition.

Correlation between degree of bradycardia and IKF shown in this cohort may be partly explained by hemodynamic changes due to increased parasympathetic activity and low cardiac outflow. In our study, no correlations between minimal HR and BMI, or between FT3 and BMI were seen. Median BMI percentile in this cohort was low but 40% of the AN cohort had a BMI percentile value above 40, reflecting the increased incidence of atypical AN, in which significant weight loss within the normal range occurs.^28^ In addition, binging/purging subtype of AN may be more commonly associated with reduced kidney function.^5^ As this study was retrospective, we could not obtain data on either weight loss rate or the subtype of eating disorder.

### Limitations

Given the retrospective nature of this study, there was not sufficient information to fully characterize IKF associated conditions, including urine analysis, electrolyte abnormalities and kidney imaging. In addition, we had to approximate height data in part of the cases group. Serum cystatin C levels were not available. We could not obtain data on weight loss rate.

## Conclusions

In this study, IKF in patients with AN is common and worsens during hospitalization. SCr values may underestimate the degree of IKF. Patients with IKF had lower minimal HR and FT3 and longer hospitalization, without differences in admission BMI. Therefore, IKF may be a better marker of AN severity.
